# Climate change and health in the Sahel: a systematic review

**DOI:** 10.1098/rsos.231602

**Published:** 2024-07-17

**Authors:** Daniel Acosta, Amadou Barrow, Idrissa Saidou Mahamadou, Victoria Simoni Assuncao, Mary E. Edwards, Sarah L. McKune

**Affiliations:** ^1^ Department of Environmental and Global Health, College of Public Health and Health Professions, University of Florida, Gainesville, FL, USA; ^2^ Sahel Research Group, University of Florida, Gainesville, FL, USA; ^3^ Center for African Studies, College of Liberal Arts and Sciences, University of Florida, Gainesville, FL, USA; ^4^ Department of Epidemiology, College of Public Health and Health Professions, University of Florida, Gainesville, FL, USA; ^5^ Department of Sociology and Rural Economy, Faculty of Agronomy, Abdou Moumouni University of Niamey, Niamey, Niger; ^6^ Department of Geography, College of Liberal Arts and Sciences, University of Florida, Gainesville, FL, USA; ^7^ Health Science Center Libraries, University of Florida, Gainesville, FL, USA

**Keywords:** Sahel, nutrition, West Africa, climate change, vector-borne diseases, food security

## Abstract

The Sahel region is projected to be highly impacted by the more frequent hazards associated with climate change, including increased temperature, drought and flooding. This systematic review examined the evidence for climate change-related health consequences in the Sahel. The databases used were Medline (PubMed), Embase (Ovid), Web of Science (Clarivate) and CABI Global Health. Hand searches were also conducted, which included directly engaging Sahelian researchers and hand-searching in the African Journals Online database. Of the 4153 studies found, 893 were identified as duplicates and the remaining 3260 studies were screened (title and abstract only) and then assessed for eligibility. A total of 81 studies were included in the systematic review. Most studies focused on vector-borne diseases, food security, nutrition and heat-related stress. Findings suggest that mosquito distribution will shift under different climate scenarios, but this relationship will not be linear with temperature, as there are other variables to consider. Food insecurity, stunting (chronic malnutrition) and heat-related mortality are likely to increase if no action is taken owing to the projected impact of climate change on environmental factors and agriculture. Seventy-one per cent of manuscripts (*n* = 58) had first authors from institutions in North America or Europe, of which 39.7% (*n* = 23) included co-authors from African institutions.

## 1. Introduction

Extensive research has been conducted on the potential impact of climate change on human health. The conclusions of most studies and systematic reviews suggest that climate change will have a net negative effect on overall health [1]. The specific impacts of climate change vary significantly by context, with the poorest countries likely facing more significant challenges in terms of health and social development, prompting calls to action and more research on climate change and global health [2,3]. In regions where extreme heat, water shortages and air pollution threaten human health, climate change is positioned to amplify these issues and to compound (and be compounded by) other challenges, such as population displacement and shifts in infectious diseases [4]. In addition to the potential impact on human health, agricultural production is likely to face significant and complex challenges owing to climate change, a reality that will undoubtedly threaten food security in low-income countries [5]. This threat is particularly worrisome in areas already chronically food-insecure such as the West African Sahel [6].

The Sahel, an agroecological zone that borders the Sahara Desert to the north and the savannah grasslands to the south, is characterized by seasonal rainfall patterns and high-temperature fluctuations and faces several concurrent challenges. Given that most people in the Sahel depend on agriculture for their livelihoods, the impact of climate change in the region is of great concern to the international community [7]. Health, education and food systems have yet to meet the demands of the region’s rapid demographic expansion [8]; estimates suggest that the population of the Western Sahel will double to around 200 million by 2050 [9]. The Sahel currently experiences high levels of food insecurity, infectious disease and forced displacement, some of which have been caused by—and all exacerbated by—an increase in the frequency and severity of extreme events, including heatwaves, droughts, floods and storms [7]. The COVID-19 pandemic brought additional temporary stress, testing the resilience of weak health systems in the Sahel, which have long suffered from governance, environmental and economic constraints [10]. In addition, outbursts of localized violence from insurgencies and terrorist groups over the past decade have further threatened the security and livelihood strategies of the population, with fears that climate change could further exacerbate these impacts [11]. There are also severe financial constraints on climate change mitigation and adaptation strategies; countries in the Sahel and across West Africa need additional support, including securing external funding and technology transfers, to meet their climate adaptation goals [12].

Research on the health risks associated with climate change and climate-related hazards in the Sahel is crucial for prioritizing and developing effective strategies and policies. Research initiated in Africa and led by academics on the continent would be particularly important, given the nature of the type of research questions and what might be explored when coming from an ‘in’ group [13]. There are, however, significant challenges in African-led academic research. The continent lags behind the global average of 1.7% of GDP invested in research, with only 0.47% of the GDP destined for research [14,15]. Additionally, research led by African institutions and universities often faces additional barriers in the indexing and publishing process [16–18]. This systematic review aims to describe the current state of knowledge of the impacts of climate change on human health in the Sahel. The review deliberately attempts to include Africa-produced research and knowledge and as such seeks to quantify the involvement of African institutions in the current state of knowledge about climate change and human health.

## 2. Methods

This systematic review was designed to analyse all peer-reviewed published literature that described research exploring climate change or its hazards on human health, broadly conceived, in the francophone Sahel. The countries included in the review are as follows: Burkina Faso, Chad, Mali, Mauritania, Niger and Senegal. Our selection of countries is based on two main criteria: first, they are all francophone and share a colonial past, and second, they are outlined by scholars as ‘the human Sahel’ owing to the herder–farmer interactions, agricultural systems and their ranking in the UN human development index [19]. Given the scope of this systematic review and its focus on health as an outcome, the following databases were included in the search strategy: Medline (PubMed), Embase (Ovid), Web of Science (Clarivate) and CABI Global Health.

Table 1 shows the model in which the search strategy for each database was created, using PubMed as an example, with the search terms used (both keywords and subject headings) in each category. Search terms varied slightly depending on the standardized keywords and features (e.g. MeSH Heading for PubMed, Emtree for Embase) for each database. The complete search strategies for each database can be found in the electronic supplementary material. The list of databases searched was compiled jointly by M.E.E. and D.A. D.A. created the search strategies in consultation with M.E.E., who served as the methodological expert on the review, guided in search development and execution and provided advice regarding proper systematic review procedures.

**Table 1.** Overview of search strategy.

concept	health	climate	geographical location
keywords	health, diarrhoea, pneumonia, vector, viruses, maternal mortality, maternal nutrition, food security, mental health, waterborne diseases, parasitic diseases, malaria, zoonoses, zoonosis, zoonotic infections, malnutrition, kwashiorkor, starvation, infants, childhood diseases, cholera	floods, drought, climate change, desert climate, tropical Africa, semiarid climate, water, environment, dust, wind, greenhouse effect, sea level, rain	Sahel, Burkina Faso, Chad, Mali, Mauritania, Niger, Senegal, West Africa
subject headings (MeSH)	health, global health, dysentery, pneumonia, vector-borne diseases, virus diseases, maternal health, food security, mental health, waterborne diseases, parasitic diseases, zoonoses, malnutrition, kwashiorkor, starvation, severe acute malnutrition, infant nutrition disorders, child nutrition disorders, cholera, one health	climate change, floods, droughts, desert climate, tropical climate, natural resources, water resources, wind, dust, environment, global warming, sea-level rise, rain	Niger, Burkina Faso, Senegal, Mauritania, Mali, Chad, Africa Central, Africa Western

**Figure 1.**
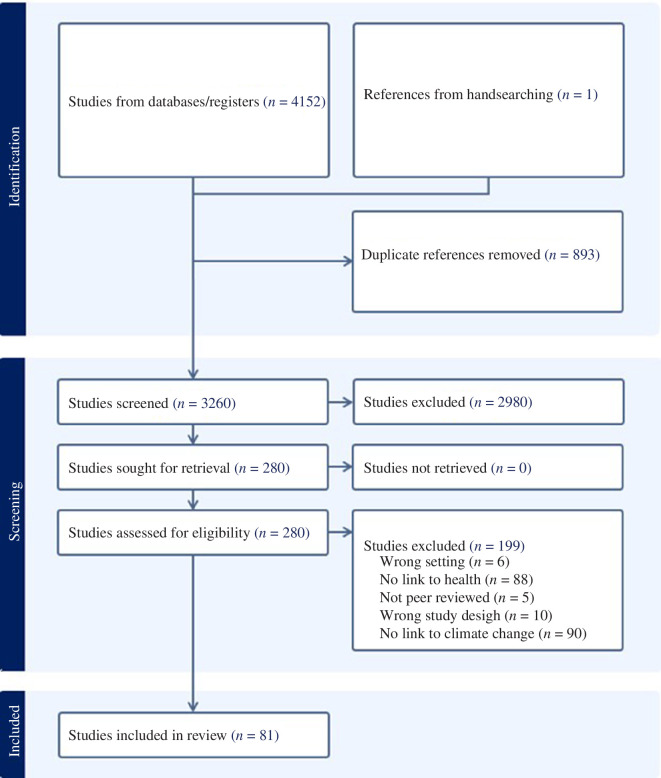
PRISMA diagram.

Screening of articles was based on title and abstract, using the following inclusion criteria: (i) peer-reviewed original research article; (ii) conducted in or about one or more of the following countries: Burkina Faso, Chad, Mali, Mauritania, Niger or Senegal; (iii) present/examine at least one health outcome (physical, mental and social well-being included), directly or indirectly linked to climate change or its associated hazards; and (iv) be written in English or French. Our search strategy had no date restrictions, and all searches were performed on 9 November 2022; articles published or indexed after this date are not included. As shown in figure 1, a total of *n* = 4152 studies were obtained from the databases, and one study [1] was included by hand-searching. The hand-searching was conducted by I.S.M. and D.A., who searched the *African Journals Online* (AJOL) database, looked for articles written by authors working in the region, and used academic WhatsApp groups and email communications with scholars to inquire about the existence of published articles that could fit the review. I.S.M. led inquiries in the Sahel, both via email and WhatsApp, to ask other researchers within his social and professional network about publications that could fit the scope of the systematic review. WhatsApp is a globally widely used messaging app, which, in many countries, such as those within the scope of the research, is one of the most common forms of communication between academics and practitioners. I.S.M. sent several messages to individuals and groups inquiring about their knowledge of existing peer-reviewed literature suitable to be included in the systematic review. D.A. led the hand search in AJOL, where different combinations of the terms in table 1 were searched. This process yielded one [1] article not previously identified that fit all inclusion criteria. After completing all searches, the research team used the software COVIDENCE, which identified 893 duplicates. A total of 3260 manuscripts were screened (title and abstract only) by two of the authors (D.A. and A.B.). From this initial review, there were 207 articles for which D.A. and A.B.’s screening were in conflict. Other authors resolved conflicts (S.L.M. resolved 88, and I.S.M. resolved 119). After conflicts were resolved, 280 manuscripts were fully screened by D.A. with the support of S.L.M., of which 199 were excluded as they did not meet the inclusion criteria. A total of 81 studies were included in the review.

D.A. led the analysis of these articles with the support of I.S.M. and V.S.A. The researchers used content analysis [20], and the data were coded using a *priori* coding [21]. The categories coded were as follows: geographical coverage, scale of study (e.g. community-level, country-wide), study setting (urban, rural, combination of both), health variables, method to estimate health variable, climate variable, method to estimate climate variable, primary outcome of the study, country of the institution of the first author, whether institutions of any co-author were based in Africa, language of publication and the year of publication. The geographical location of the studies was categorized by extracting information from the methodology of the selected studies to identify the countries where the research was carried out. This variable only considered research done in a specific country or a specified combination of countries (e.g. comparing two countries). If a study was conducted at a global, continental or regional scale without highlighting findings from a specific Sahelian country, but making reference to the region, then the study was not assigned a ‘country location’. The health topics were coded by identifying the primary variable to estimate a health outcome. For instance, if a study used variables like stunting or wasting, this would be considered a nutrition study. When a study used surveys (e.g. Household Food Insecurity Access Scale, Food Insecurity Experience Scale) or other instruments (focus groups, secondary data) to measure food availability but did not collect biological samples or anthropometric measurements, these studies were classified as ‘Food Security’ studies. To code collaboratively, given that researchers were based in the USA and Niger, the research team used Google Sheets to code the information obtained.

## 3. Limitations

Data from the AJOL database were only partially extracted, as it was not possible to extract all results. Results were truncated after 100 articles were shown, and the research team had no success resolving this issue; hence, some results from this database were undoubtedly excluded since extraction was not possible. Additionally, the relationship between climate change and food security in the region is deeply interconnected with economics, as most of the region’s population depends on agriculture for their livelihoods. Economic impacts at a broad scale, which can be caused by climate hazards, can influence food security and other health outcomes. We did not include studies that solely looked at economic impacts unless they used instruments to measure food security or other health outcomes in their methods. Furthermore, studies of this type are limited to those that are published in peer-reviewed literature. Research on the Sahel is generally limited, but the number of published peer-reviewed articles is further constrained by publication practices and procedures long dominated by Western institutions that have always come with a price tag that further limited African scholars from engaging. The costs associated with knowledge production and access, such as institutional subscriptions or open access fees, have limited researchers in the Sahel from publishing their own work. There is rigorous scientific research being done across the Sahel, but the barriers to publication significantly limit those that reach an international audience, especially in journals that are indexed in the most commonly used databases.

## 4. Results

The most common research topic identified on health was malaria and other vector-borne diseases (*n* = 19), followed by food security (*n* = 17), nutrition (*n* = 14) and heat stress/waves (*n* = 7), as seen in table 2. These four topics cover over 70% of the studies that met the inclusion criteria. A detailed table with information and classifications for each of the studies can be found in the electronic supplementary material.

**Table 2.** Authorship distribution.

continent	number of publications by the continent of the first author’s institution	number of publications that included co-authors from an African institution
Europe	32 (40%)	20 (63%)
North America	26 (32%)	3 (12%)
Africa	21 (26%)	21 (100%)
Asia and Oceania	2 (2%)	2 (100%)

### 4.1. Malaria and other vector-borne diseases

Twelve studies focused on malaria, while seven focused on other vector-borne diseases. When it comes to future projections of malaria transmission in the Sahel in relation to climate change, six studies suggest that an increase in temperature might reduce the incidence of malaria [22–27] as some of the most important vectors (e.g. *Anopheles gambiae*) are not suited for warmer conditions [28]. One study found that malaria has been increasing in the Sahel since the droughts of the 1970s and 1980s as it moved from a dryer to a wetter climate [29], which corresponds to the findings of five studies that identified rainfall and river height as a risk factor for malaria [22,26,30–32]. Only one study focused on urban malaria. That study did not focus on malaria transmission, however, but rather on self-medicating practices among febrile children in a large urban area (Dakar). This study showed that people living in areas with high levels of environmental threats (dense vegetation and stagnant water) are more likely to seek medical care rather than self-medicating owing to their perceived environmental risk [33].

When it came to other vector-borne diseases, six of the seven studies focused on other diseases transmitted by mosquitoes (e.g. Rift Valley fever or dengue), with four of those at a large scale (global, continent or West African level) [34–37]. The two other studies were conducted in rural Senegal [38,39]. The findings of these studies vary, with some suggesting that higher rainfall could increase the presence of several mosquito species [34,36,39]. Other studies, however, hypothesize that higher temperatures in the Sahel could reduce the suitability of specific mosquito species, with species like *Culex quinquefasciatus*, *Aedes albopictus* and even *Aedes aegypti* (in the most extreme warming scenarios) becoming unable to function as vector of diseases [35,37]. Owing to the complex relationship between mosquito demographics and climate variability, one of the studies suggests that the relationship between climate change and mosquito populations in the Sahel will be nonlinear [38]. One study focused on Lassa fever transmission, with projected increased spillover events by 2070 driven by climate change and human population growth [40]. The studies proposed that certain effects of climate change, such as higher temperatures, could lead to a decrease in malaria cases in the Sahel, while others, such as rainfall, could result in increased suitability for vector-borne diseases. This suggests that the effects of climate change on vector-borne diseases in the Sahel are not linear, and gaps remain in our current understanding of the potential impacts of climate change on the health of the population in the region.

### 4.2. Food security

In the studies regarding the impacts of climate change on food security, six highlighted the role of livestock. Four of the studies mentioned that livestock ownership is positively associated with food security [41–44], while the other two identified livestock ownership as the means (traction, organic fertilizer) to enhance farming techniques that were positively associated with food security [45,46]. Other predictors of food security for populations impacted by climate change or climate hazards were diversification [46,47], intensification [43], household market orientation [43], use of fertilizer [45], use of other climate adaptation strategies [48,49] and members of the household engaging in non-farm activities [45,46]. One of the studies found no relationship between food security and a household’s engagement in non-farm activities [49]; however, the authors hypothesize that this is owing to the underdevelopment of non-farm activities available in the study site. Overall, studies found that climate change threatens food security in the region and that several potential adaptation strategies could mitigate the impact of climate change on food security; however, these vary widely depending on the agroecological and social context..

### 4.3. Nutrition

There were 15 studies in which climate change and nutritional outcomes (e.g. stunting, wasting) were studied in the Sahel. Most of these studies (*n* = 11) relied solely on secondary data. From the 11 studies that used secondary data, 8 obtained the data to estimate their nutritional variables from the Demographic Health Surveys (DHS) [50–57]; the other studies used secondary data from the Nouna Health and Demographic Surveillance System [58] and from impact evaluation and monitoring surveys of development programs [59,60]. Utilizing existing datasets, such as the DHS data, which provides important insights, comes with many limitations and risks. Data can be outdated, and the quality cannot be assessed by the researchers utilizing it. Even though the topic of nutrition is among those most studied in the region, most of the data used come from the same source, limiting the scope of understanding.

Projected climate variations are associated with adverse outcomes in children’s nutritional status, with studies finding an association between high temperatures and malnutrition (both acute and chronic malnutrition) [50,53]. For instance, increased exposure to temperatures of 35°C can reduce height-for-age standardized scores by a fifth of a standard deviation. A study also found that heat exposure in Burkina Faso is associated with a reduction in breastfeeding time, which could potentially reduce the odds of exclusive breastfeeding during periods of elevated temperatures [61]. The findings suggest that heat exposure alone can have detrimental effects on child nutrition, which are then compounded with other negative health outcomes that come from constant exposure to elevated temperatures. Studies also link rainfall shocks to stunting [53,59,62]. A short communication reporting preliminary results from a study in Burkina Faso also found potential links between rainfall variability and increased rates of stunting [63]. Other climatic shocks, such as greenness anomalies and drought, have also been linked to stunting [51,52,56,60,64]. These findings suggest that potential climate hazards and long-term climatic changes (e.g. shorter, more intense rainy seasons and higher temperatures) could have compounding detrimental effects on child nutrition. While studies have previously identified environmental links to malnutrition, variability in rainfall and temperature demonstrates a distinct link between climate change and nutritional outcomes in the Sahel.

### 4.4. Heat stress

Seven studies in the systematic review focus on the direct effects on human health of increasing temperatures. Three of the studies focused on projected temperature increases, two on a global scale [65,66] and one specific to the Sahel [67]. All three studies found that the Sahel will be exposed to extreme temperatures under different climate scenarios. The study that focused on the Sahel used three scenarios: RCP 2.6, RCP 4.5 and RCP 8.5. The most optimistic scenario (RCP 2.6) showed that 15 day heat waves will become the norm by the end of the century in the Sahel. The intermediate scenario (RCP 4.5) showed potential increases in temperature of about 3.5°C by the end of the century. Under the RCP 8.5 scenario, the temperature is expected to be +5°C by 2100, and the spring season in the Sahel could be considered a permanent 3 month heatwave, with a few models suggesting this could occur as early as 2070 [67]. West Africa is identified as being at the highest level of experiencing a dramatic increase in heat health risks, with potentially large portions of the population being exposed to severe heat stress [65,66]. Some of the studies extracted found an association between higher temperatures and mortality [68,69], as well as an increase in mortality owing to cardiovascular diseases [70]. A pilot study in Burkina Faso found temperature variations between different types of housing units, with some types currently exceeding extreme danger levels of heat exposure [71]. Current literature shows a clear relationship between climate change impacts and increased exposure to hazardous temperatures in the Sahel. The heat-associated increase in mortality and disease (e.g. cardiovascular diseases) has been highlighted and is among one of the most consistent messages in the literature.

### 4.5. Health perceptions and mental health

Seven of the studies extracted found an increase in adverse self-reported health outcomes or an increase in the perception of health risks [72–77]. A study in Burkina Faso found that perceptions of climate impacts on health included food security, vector-borne diseases, water-related illnesses and respiratory diseases [73]. A study in Pikine, Senegal, found that floods in urban areas have also been associated with perceptions of negative health outcomes, with households identifying increases in malaria, skin diseases and diarrhoea when flooding occurred [75]. A similar study used a multidimensional vulnerability index specific to drought that included health threats [78]. Three studies focused on mental health and well-being, with results showing increasing mental distress issues caused by shocks and cultural loss owing to climate change [79–81]. These findings underscore the link between environmental hazards and perceptions of health risks, which was also highlighted in one of the studies about malaria cited above, where people with higher exposure to hazardous environments were more likely to seek medical attention in the case of febrile events [33]. While there are very few studies in the Sahel about mental health and climate change [79–81], all of them found negative effects on mental health.

### 4.6. Meningitis and particulate matter 2.5 exposure

Five studies focused on meningitis indicate that climate variability, especially during the dry season owing to high dust loads and low humidity, is associated with meningitis outbreaks [82–86]. Two studies that included the whole African continent found that infant mortality in West Africa and the Sahel owing to exposure to particulate matter (PM) 2.5 was likely to increase owing to climate change [87,88]. Even though the literature on the topic is scarce, the directionality of the current literature shows that environmental conditions for meningitis and exposure to PM 2.5 are likely to increase as shifts in climate provide more favourable conditions.

### 4.7. Water, sanitation and hygiene: cholera and diarrheal diseases

Only two studies, both in Burkina Faso, focused on water, sanitation and hygiene practices in the context of climate change and health impacts in the Sahel [89,90]. Women in Burkina Faso faced more significant risks and difficulties than men when faced with limited water availability during the dry season. Furthermore, women from the Peul ethnic group were found to use water of poorer quality owing to their distance from water points, potentially exacerbating health risks [89]. The other study focused on the potential health impacts of water harvesting and storage, concluding that in some cases, this practice (a widely promoted climate change adaptation) could potentially increase transmission of waterborne illnesses owing to poorly planned and managed storage [90].

Three studies focused on diarrhoeal diseases, and one focused on cholera outbreaks. One of the studies suggests that higher temperatures and rainfall were associated with higher diarrhoeal incidence, with temperature being a stronger predictor of diarrhoea in rural settings and rainfall in urban areas [91]. The other two studies observed a high prevalence of *Escherichia coli* and reported a peak in diarrhoeal diseases during the rainy season [92,93]. Regarding cholera outbreaks, one study suggests that the cholera epidemic of 2005 in Senegal was exacerbated by floods, which could become more recurrent in the context of climate change [94]. These findings suggest that exposure to pathogens that cause diarrhoeal disease is likely to increase if extreme climate events such as drought and flood increase. Droughts can potentially lead to more dependency on water harvesting and storage (which, if done incorrectly, can increase exposure to pathogens) and limit access to cleaner sources of water. Floods on the other hand have been linked to outbreaks of diarrhoeal diseases.

### 4.8. Geographic distribution of research, authorship and involvement of African institutions

**Figure 2.**
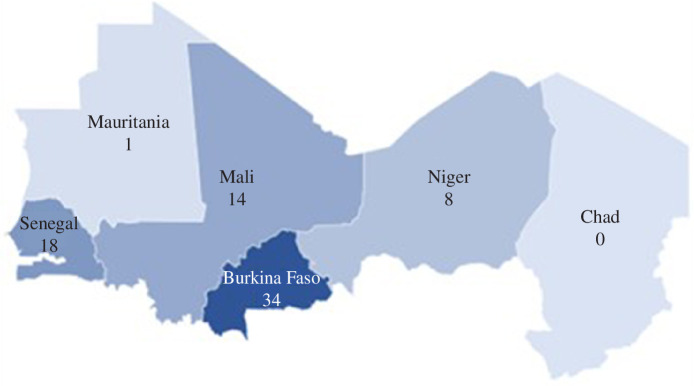
Number of studies per country.

A total of 15 studies were found at the global, continent or regional level. These studies were excluded from the variable ‘country location’. Figure 2 shows the number of publications that fit the inclusion criteria and were carried out at a country-specific level. No studies were explicitly conducted in Chad, and only one was found for Mauritania. Most of the studies included were conducted in Burkina Faso (*n* = 38), Senegal (*n* = 18) and Mali (*n* = 14).

Most of the publications had first authors based in institutions in Europe (*n* = 32), followed by North America (*n* = 26), Africa (*n* = 21) and Asia/Oceania (*n* = 2). Table 3 shows the number of publications organized by the continent of the first author’s institution and the percentage of those manuscripts that had co-authors from an African institution. Seventy-one per cent (*n* = 58) of the manuscripts included in the systematic review had first authors affiliated with European and North American institutions. Of those, only 39.7% (*n* = 23) included co-authors from an African institution. The inclusion of co-authors from African institutions varied depending on the continent. Sixty-three per cent (*n* = 20) of manuscripts with first authors from European institutions had co-authors from African institutions, which is higher than those from North America (12%, *n* = 3). 26% (*n* = 21) of the publications in the systematic review had first authors from African institutions, of which 52% (*n* = 11) had co-authors from Europe or North America.

**Table 3.** Number of studies by topic.

topic	number of studies
malaria and other vector-borne diseases	19 [22–27,29–40,95]
food security	17 [41–49,96–103]
nutrition	15 [50–64]
heat stress/waves	7 [65–71]
health perceptions	7 [72–78]
meningitis	5 [82–86]
diarrhoeal disease and cholera	4 [91–94]
mental health and well-being	3 [79–81]
WASH	2 [89,90]
exposure to PM 2.5	2 [87,88]

WASH, water, sanitation and hygiene.

**Figure 3.**
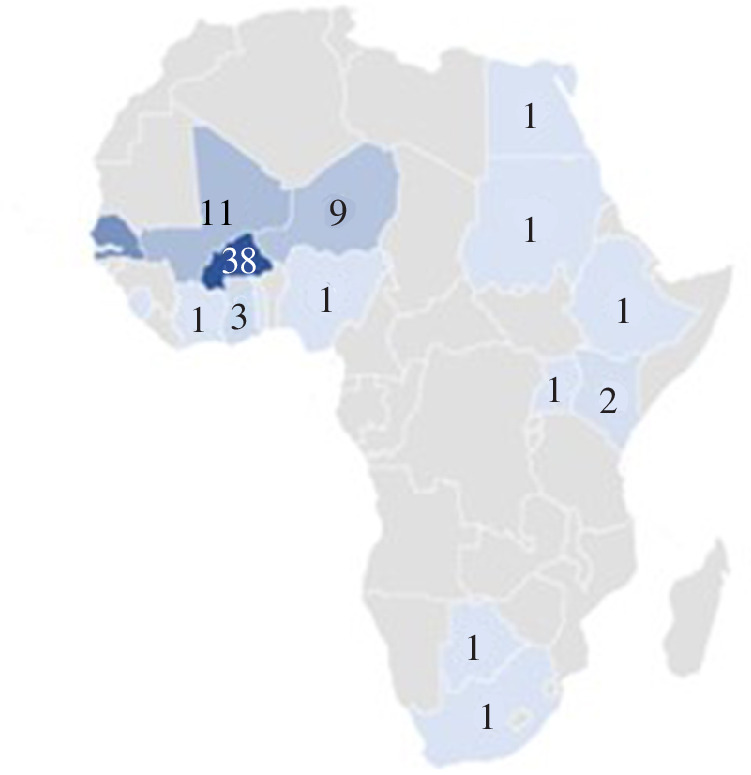
Distribution of authorship affiliations in African institutions.

Most authors and co-authors from African institutions were based in Burkina Faso (*n* = 38), followed by Senegal (*n* = 22), Mali (*n* = 11) and Niger (*n* = 9). The institution with the highest number of authors was the Centre de Recherche en Santé de Nouna in Burkina Faso (*n* = 8), followed by the Université Cheikh Anta Diop in Senegal (*n* = 6) (figure 3).

## 5. Discussion

While the results show that important gaps in the literature exist when it comes to the impacts of climate change on health in the Sahel, the findings also signal how climate change is currently impacting and threatening health in the region. Given the importance of rainfed agriculture and pastoral livestock systems, the nexus of climate change, agriculture and health must be an important policy and research consideration for all stakeholders. If the occurrence of extreme climate events continues to increase, the risk of stunting, wasting and increased household food insecurity will increase. There are, however, challenges in quantifying the exact impacts of climate change on health. For instance, few studies in the systematic review studied the relationship between climate change and mental health [79–81]. However, some studies link food insecurity (not necessarily caused by climate change) with adverse mental health outcomes in Africa [104]. The ramifications of increased climate-induced food insecurity could thus indirectly cause or exacerbate other adverse health outcomes such as mental health. The intricate relationship between climate change and food, health, agricultural and economic systems makes climate change impact projections challenging. This study excluded publications that solely used economic models without using health-related and/or food security data; however, climate change impacts on the economy of Sahelian countries are likely to also impact health. In addition, other variables that are external to the Sahel and unrelated to climate change could have effects that exacerbate the impact of climate change on health. For instance, geo-political conflicts in Eastern Europe have contributed to declining supplies of fertilizer in the Sahel [105]. Given that fertilizer use is associated with food security in the context of climate change, declining supply could have a negative impact on food security [45,46].

In terms of vector-borne diseases, a reduction in the range of malaria vectors throughout the Sahel is projected under increased temperatures by several studies [22–27]; however, there are very few studies in the region on the potential dynamics of other vector-borne diseases [34–39]. Even though a decrease in suitability for malaria vectors is desirable, it is unclear whether this could yield a net-positive outcome for health in the region. There is a need for more studies to fully understand the disease vector dynamics under different climate change scenarios. Studies at the community level are important for the design of vector control strategies in the future. Studies done at the continental level might project an overall decrease in vector suitability; however, some places in the region might have conditions in which higher rainfall leads to an environment that is favourable for disease vectors [34,36,39]. Other factors might also impact the transmission of vector-borne diseases, such as rapid urbanization, which could lead to an increase in suitable vector habitats for *Aedes* mosquitoes, increasing the risk of dengue, Zika, chikungunya and other arboviruses [106].

All of the studies regarding heat stress raised important concerns about increased mortality rates associated with heat waves and heat stress in the Sahel [65–71]. Increased exposure to hazardous temperatures could increase mortality and the prevalence of cardiovascular diseases. Furthermore, other important ramifications of increasing temperatures and heat waves can ultimately impact health. If temperatures increase, agricultural practices, including livestock production, might become more challenging and could add additional stress to water systems. Increased heat exposure has also been linked to decreased breastfeeding times [69] which could yield negative nutritional outcomes as recent studies have shown an urgent need to improve infant feeding practices [107]. The results from the systematic review show that climate change currently poses and will continue to pose significant challenges to health systems in the Sahel, but also that there are substantial gaps in the current research.

This study found that there are regional disparities regarding research fields and their geographical focus. Health issues such as heat stress, mental health and other non-communicable diseases and their relation to climate change remain largely unstudied in the region. Given that the Sahel has been identified as a high-risk region for the impacts of climate change [7], the gaps in the literature identified in this systematic review highlight the barriers to creating data-driven policies. Geographically, Chad and Mauritania currently have more significant research gaps. Furthermore, although there were several studies of vector-borne diseases, food security and nutrition, many of these studies, particularly nutrition studies, relied on secondary data at a macro-level, which increased the limitations and limited the meaningfulness of the results at the local level. There is a need for more localized research on the impacts of climate change on health across the region.

The systematic review process also highlighted the lack of involvement of authors from African institutions in the publication process, with just over half the studies (56.7%) including a co-author from an African institution and only 26% of studies in the review having first authors from African institutions. This, however, could be partly owing to the limitations of the current bibliometric system, which has high entry barriers for African journals and researchers [16]. The systematic review also showed high disparities in the inclusion of authors from African institutions among North American and European institutions. Only 12% of manuscripts with first authors from North America had co-authors from African institutions, a stark difference compared to studies led by authors from European institutions, which had co-authors from African institutions in 63% of the studies found. A systematic review of global health publishing practices found similar results, where lower representation from African institutions occurred when collaborating with top US-based universities [108].

## 6. Conclusion

Climate change clearly poses a threat to health and health systems in the Sahel, although understanding the precise nature of the threats and the interactions among various factors requires further investigation. Results from the systematic review show that malaria, food security and nutrition are, among published studies, the most researched climate-related health topics in the region. A limitation of nutrition studies is that they rely on secondary data. And though these studies offer valuable insight, they have more significant limitations and less applicability to the current local context. Climate change impacts on agriculture that threaten food security and nutrition are of great concern, and strategies to enhance food security have been highlighted in some studies in the systematic review. The literature indicates that possession of livestock, fertilizer use and household market orientation were some of the adaptation strategies that most increased food security, although these vary depending on the agroecological and social context. Findings also suggest that heat waves and extreme heat conditions substantially threaten the region’s health. Few studies were found in the systematic review that linked climate change to mental health, waterborne illnesses and meningitis outbreaks [79–81,87–90]. Those few that did signalled that projected climate change or past climate trends could worsen outcomes related to each of these health issues. In summary, despite projections of a significant impact of climate change in the region, research into the health consequences of climate change in the Sahel remains widely understudied, and there are important disparities in research outputs across the region, with larger research gaps in Chad and Mauritania. The results of the study of authorship suggest that North American institutions should increase their collaborative research efforts to foster authorship opportunities with researchers in African institutions.

## Data Availability

This article has no additional data. Supplementary material is available online [109].
